# Prevention of Typhoid Fever

**Published:** 1889-12

**Authors:** J. N. M’Cormack

**Affiliations:** Secretary; Office of the State Board of Health, Bowling Green, Ky.


### PREVENTION OF TYPHOID FEVER.

The following circular has been issued by the Kentuckey State Board of health:

Office of the State Board of Health,
Bowling Green, Ky., August 12, 1889.

*To the Health Officials and People of Kentucky:*

This Board desires to call the earnest attention of our health authorities and people to the gradually increasing prevalence of and mortality from typhoid fever, and to the growing importance of a constant resort to the methods which modern scientific researches have suggested for the prevention of this disease.

These preventive measures are of the more importance to the State because directed against a disease especially prevalent and fatal among persons in the prime of life, who contribute most to the public wealth and prosperity. Considered purely as an economic problem, the feature of it probably least thought of by most people, the importance of this disease can scarcely be over-estimated. Statistics show that ten persons are sick for every one that dies of this disease; and to say nothing of the cash value to the State of those who die every year, and it is conceded that the State has no more valuable property than that represented in its vigorous population, the loss of time and labor, and the necessary cost incurred in attention to those who finally recover, makes an annual tax upon our people of startling proportions.

Typhoid fever is probably the most preventable of all diseases, not even excepting smallpox. It is now known that like cholera and dysentery, the germ or specific cause of this disease is contained in the discharges from the bowels of those sick of it, and that while other methods of introducing the poison into the system are possible it most generally gains entrance through the medium of an infected water supply—usually the use of well-water polluted by fecal matter. This may be direct, from drinking such water, or indirect, as by using milk or other articles of food or drink from cans or vessels washed in it. Ice from an infected source is also dangerous, since it has been proven that freezing does not destroy the infective principle.

While water from all sources of supply is liable to contamination, well-water is especially so, whether located in city, town, summer watering-place, or country. Thus, out of three hundred and fourteen cases occurring in Louisville in 1884, two hundred and ninety-eight of the persons used well-water habitually, and some of the other sixteen did so occasionally. In the now famous epidemic at Plymouth, Pennsylvania, involving the sickness of 1,104 persons, the death of 114, and an actual outlay in money of $67,100.17, the out break was traced to the use of water polluted by the fecal discharges of one imported case of the disease. Facts no less convincing might be multiplied indefinitely if space permitted. In a smaller way they are common in the experience of most physicians in active practice.

Usually the wells are sunk near the kitchen, and in dangerous proximity to the privy and other sources of contamination. The well draws its supply from an inverted cone, having its apex at the bottom of the well and its base at the surface of the ground. In dry seasons this base is often extended until the well becomes a receptacle for the more or less perfectly filtered filth from all the sources found in the average back-yard, and the water, often sparkling in its apparent purity, becomes a culture fluid for any disease germs finding their way into it.

Two methods of prevention, having the same general object in view, are to be recommended. The first involves the thorough disinfection of all discharges from the bowels of typhoid fever patients. This is best done by the use of a solution of chloride of lime, eight ounces to the gallon of water, using a quart of this solution for each discharge, and allowing it to stand in the vessel at least one hour before emptying. A solution of corrosive sublimate, two drams to the gallon of water, will answer the same purpose, but requires to remain longer in contact with the material to be disinfected. Bed and body linen soiled by such patients should be disinfected by the use of the same solution or by boiling.

The second method relates to avoiding the use of suspicious water, and especially well-water, and, where this can not be done, to boil such water before it is used for drinking purposes. In the absence of a pure and well-guarded public water supply, properly stored cistern water is probably open to least objection.

The effectual practice of these methods will require intelligent care and some expense, but it is confidently believed that their general adoption would result in the practical disappearance of a disease which is not only a disgrace to our civilization, but an annual scourge and tax upon the people of Kentucky, in comparison with which yellow fever and cholera sink into insignificance.

J. N. M'CORMACK, M. D., Secretary.

